# Uncovering the emotional aspects of working on a clinical trial: a qualitative study of the experiences and views of staff involved in a type 1 diabetes trial

**DOI:** 10.1186/1745-6215-16-3

**Published:** 2015-01-07

**Authors:** Julia Lawton, Jackie Kirkham, David White, David Rankin, Cindy Cooper, Simon Heller

**Affiliations:** Centre for Population Health Sciences, University of Edinburgh, Teviot Place, Edinburgh, EH8 9AG UK; Clinical Trials Research Unit, University of Sheffield, 30 Regent Street, Sheffield, S1 4DA UK; Academic Unit of Diabetes, Endocrinology and Metabolism, University of Sheffield, Beech Hill Road, Sheffield, S10 2RX UK

**Keywords:** Qualitative research, Trial staff, Emotional labour, Emotion work, Type 1 diabetes, Randomised controlled trial

## Abstract

**Background:**

The perspectives and experiences of trial staff are increasingly being investigated as these can be used to improve recruitment, adherence to trial protocols and support given to future staff. We interviewed staff working on a type 1 diabetes trial in order to aid interpretation of trial findings, inform recommendations for the rollout of the treatments investigated and provide recommendations for the conduct of future trials. However, our interviews uncovered aspects of trial work erstwhile unrecognised or underreported in the trials literature, and it is these which form the focus of this paper.

**Methods:**

In-depth interviews were conducted with (n = 18) staff, recruited from seven centres, who were involved in recruitment and trial delivery. Data were analysed thematically.

**Results:**

Alongside logistical and practical issues which made trial work challenging, staff often talked spontaneously and at length about how trial work had affected them emotionally. Staff not only described the emotional stresses arising from having to meet recruitment targets and from balancing research roles with clinical responsibilities, they also discussed having to emotionally manage patients and their colleagues. The emotional aspects of trial work particularly came to the fore when staff notified patients about their treatment allocation. On such occasions, staff described having to employ emotional strategies to pre-empt and manage potential patient disappointment and anger. Staff also described having to manage their own emotions when patients withdrew from the trial or were not randomised to the treatment arm which, in their clinical judgment, would have been in their best interests. To help address the emotional challenges they encountered, staff highlighted a need for more practical, emotional and specialist psychological support.

**Conclusions:**

More attention should be paid to the emotional aspects of trial work to help ensure trial staff are adequately supported. Such support could comprise: increased training for staff to improve their own and patients’ understandings of randomization, role-play to develop techniques to manage patient anger and disappointment, sharing of good practice, formalised team support with psychological input and access to specialist psychological support to troubleshoot complex emotional and ethical issues.

## Background

Research drawing upon participants’ perspectives and experiences is increasingly being undertaken as part of clinical trials to help inform recruitment, trial delivery and improve the conduct of future trials [1]. To date, the overwhelming majority of studies have focused on patients’ understandings and experiences, and how these may affect recruitment, retention and adherence to the treatments investigated during the trial [2–10]. However, the benefits of including staff perspectives have also been highlighted, with studies showing how these can be used to improve recruitment [11], adherence to trial protocols [12] and the support given to individuals working on future trials [13]. Most research undertaken with staff has focused on, and drawn attention to, barriers to recruitment [14, 15], such as busy clinics, lack of resources [16, 17], insufficient consultation with clinical staff [17, 18] or there being fewer patients meeting inclusion criteria than originally anticipated [19]. Studies have also highlighted how recruitment and trial delivery can be affected by poor understanding amongst staff about what a trial is trying to achieve [16, 20], perceived pressures to meet trial recruitment targets [13] and funding systems and financial considerations [21].

Complementing these studies, a growing body of literature has highlighted the ethical issues which can arise for trial staff due to their experiencing conflicts between research and clinical roles [22–25]. This literature has also explored how trial staff may attempt to address these role conflicts, for instance, by separating research and clinical activities and/or by inserting individualised care into the trial [23–27]; in some instances undermining adherence to trial protocols [18, 25]. More recently, the emotional impact of trial work on staff has begun to be raised and discussed, this issue having erstwhile been implicit in the trials literature [22, 28, 29]. Notably, in a recently published synthesis of findings from interviews with staff involved in recruiting to six pragmatic randomised controlled trials (RCTs), Donovan *et al*. document what they term ‘hidden challenges’ to recruitment [30]. Specifically, they highlight the emotion and discomfort staff can experience when they are confronted with conflicts between research and clinical roles and/or when they struggle with the concept of equipoise [30, 31]. In an interview study with staff involved in recruiting children to clinical trials, Shilling *et al*. likewise uncovered emotional issues that arose for staff [32]. Specifically, they found that staff could worry and feel anxious about burdening families and overwhelming them with information.

In this paper, we seek to advance the literature further by bringing the emotional aspects of trial work further to the fore. To do this, we report findings from interviews undertaken with staff involved in an RCT for people with type 1 diabetes who were responsible for recruitment and delivering the interventions investigated during the trial. The aim of these interviews was to identify issues which arose for staff during the trial which could be used to help explain trial findings. We also sought to inform recommendations for the rollout of the treatments investigated and the conduct of future trials. However, like Donovan *et al*. [30], our interviews with staff not only revealed issues which are already well recognised in the literature, such as organisational barriers to recruitment, they also uncovered unexpected and hitherto undocumented emotional challenges. Specifically, as we report in this paper, staff not only highlighted the emotional impact on themselves of undertaking work during the trial, they also described having to undertake emotion work on patients and their colleagues. In doing so, we will argue that emotional labour, a concept which is now well recognised and widely used in the nursing and midwifery literatures, has been surprisingly overlooked in the literature on trials. As a precursor to presenting our findings and discussing their implications, we will begin by briefly introducing the concept of emotional labour, the trial which formed the focus of our study and our qualitative study design.

### Emotional labour

The concept of emotional labour first came to the fore thanks to a ground-breaking study of flight attendants undertaken by Hochschild in the 1980s [33]. Defined as, ‘the induction or suppression of feeling in order to sustain an outward appearance that produces in others a sense of being cared for in a convivial and safe place’ (page 7, [33]), Hochschild showed emotional labour to be a key aspect of flight attendants’ work, one which, like members of other service sector industries, they were explicitly trained and paid to undertake [33]. Subsequent to this, this concept has been extended to understand the work of healthcare providers, principally those working in nursing and midwifery disciplines [34–36]. However, unlike Hochschild’s flight attendants, and because of heavy emphasis placed on physical labour in nursing and midwifery work [34], researchers have suggested that emotional labour remains an ostensibly invisible and undervalued aspect of the work of nurses and other healthcare providers. Hence, a key agenda of this literature has been to raise awareness of the importance and skilled nature of this aspect of healthcare providers’ roles, and to call for better training and support to be offered to staff in the future [34–36]. Such an agenda is also taken forward in this paper; albeit, in this instance, to offer better and tailored support to staff working on trials, such as the one we now go on to describe.

### The Relative Effectiveness of Pumps Over MDI and Structured Education (REPOSE) trial

The Relative Effectiveness of Pumps Over MDI and Structured Education (REPOSE) trial is a two-arm, multicentre RCT comparing two types of insulin treatment delivery for adults with type 1 diabetes provided with high-quality education - pump therapy versus multiple daily injections (MDI) [37]. At present, most patients with type 1 diabetes use an MDI regimen and the trial was developed to determine whether pumps might be a better and more cost-effective option. Trial participants were required to attend a week-long structured education course called Dose Adjustment For Normal Eating (DAFNE), so they could receive comprehensive instruction on how to accurately count carbohydrates and adjust their insulin doses accordingly [38, 39]. As DAFNE courses were originally developed for patients using an MDI regimen, an adapted course was used for those randomised to the pump arm of the trial so that they could also receive training in the use of pump technology. To be eligible for the trial, patients could not have previously used an insulin pump, attended a DAFNE course (hence patients on DAFNE course waiting lists were targeted for recruitment into the trial), or have a stated preference for one type of treatment over the other. Checklists were used to help ensure patients met all trial eligibility criteria. Patients were provided with an information sheet prior to deciding whether to take part and those taking consent were instructed to discuss key aspects of the trial to help foster patients’ understanding, including knowledge of pump therapy and the randomisation process. Consenting patients were allocated a place on one of two DAFNE courses. These courses were then randomly allocated to either pump or MDI treatment at least four weeks in advance of the first course date in the pair. After attending their pump or MDI courses, patients’ clinical care was returned to their routine healthcare providers which, in practice, were often the same staff who were responsible for trial recruitment and delivery. Clinical data for the trial are being collected at six, 12 and 24 months [37].

The trial is being conducted in eight centres in Scotland and England, most of which ran three pairs of DAFNE pump and MDI courses. The courses were normally delivered by two DAFNE educators, usually a diabetes specialist nurse and a dietitian. As well as being responsible for delivering the courses, one or more of these educators also took responsibility for recruitment in each centre, supported by a central Clinical Trials Unit (CTU). As well as undertaking a health economic evaluation and statistical analysis of the final trial findings, the CTU was responsible for overall management of the trial, including: periodic updating of the trial protocol and operating procedures; data management, randomisation and trial monitoring; providing centres with detailed reminders of trial timelines and recruitment deadlines; facilitating educator teleconferences and providing regular updates on the trial’s progress, including recruitment numbers achieved in the different trial centres.

## Methods

In-depth interviews were used in this study as they afforded the flexibility needed for staff to raise and discuss issues which were most salient to them, including those potentially unforeseen at the study’s outset. The study was informed by the principles of Grounded Theory research and entailed concurrent data collection and analysis, enabling issues and themes identified in early interviews to iteratively inform the areas explored in later ones and also sampling [40, 41].

### Recruitment and participants

In each trial centre, as already reported, courses were typically run by two DAFNE educators: a dietitian and a diabetes specialist nurse. Some centres used the same educators for all six courses, whereas others divided the work amongst their team so that different pairs of courses were run by different individuals. We initially aimed to recruit at least one diabetes specialist nurse and one dietitian from each centre to explore their recruitment and trial delivery experiences. However, after early interviews had been conducted and analysed, we decided to increase the number of nurses with whom we spoke. This was because these team members tended to have the greatest involvement in recruitment and notifying patients about the outcome of randomisation and our interviews highlighted that these aspects of trial work were particularly emotionally challenging and required complex emotional and negotiation skills.

Staff were recruited from seven centres. The eighth centre was not included as it was a reserve centre added at the end of the trial to deliver two courses only. The final sample comprised 12 nurses and six dietitians; due to staff leave we were unable to interview the dietitian in one of the centres. Participants were sent recruitment packs and written informed consent was obtained prior to the interviews.

### Data collection and analysis

Interviews were conducted between December 2012 and April 2013, and were timed to take place one to three weeks after each centre’s sixth REPOSE course had been completed. This was to avoid any risk of inadvertent contamination of the trial intervention by the qualitative questioning, and also because, at this point, we anticipated staff would have had considerable experience of trial recruitment upon which they could reflect. Staff were interviewed at their workplace. The interviews were informed by a topic guide developed in light of literature reviews, our previous experience of undertaking interviews with staff involved in clinical trials [13, 25] and revised in light of emergent findings (see above). Topics relevant to the findings reported in this paper cohered around the following areas:

-  Clinical background and training in diabetes (including years of diabetes clinical experience); current clinical roles and responsibilities.
-  Reasons for taking part in the REPOSE trial; initial understandings and expectations of the trial.
-  Duties and responsibilities on the trial; impact of trial work on routine clinical roles and responsibilities.
-  Experiences of recruiting patients into the trial (including any challenges and barriers encountered and the reasons for these).
-  Experiences of, and views about, the trial randomisation process and informing patients about their treatment allocation.
-  Contacts with patients during the trial and how these were managed to optimise recruitment and minimise attrition; perceptions of the positive and negative aspects of trial work.
-  Recommendations for how recruitment and delivery could be improved in future trials.
-  Views about the support offered and received (including from the CTU) during the trial and how this might be improved on future trials; unmet needs for information, training and support.

Interviews averaged an hour, were digitally recorded and transcribed in full for in-depth analysis.

Data were analysed thematically using the method of constant comparison [41]. This entailed individual interviews being read through repeatedly before being cross-compared to identify common issues and experiences. The analysis was undertaken by JL and JK, who wrote separate reports before meeting to discuss and reach agreement on key findings, identify emerging themes requiring more detailed exploration and to develop a coding frame. The qualitative analysis software package NVivo9 (QSR International, Doncaster, Australia) was used to facilitate data coding and retrieval. Coded datasets were subjected to further, in-depth analysis to identify sub-themes and illustrative quotations. By the time data collection was completed no new themes were emerging in the analysis [40].

The REPOSE clinical trial, including the qualitative sub-study, was granted NHS ethics approval by the North-West Research Committee (Liverpool West), approval number 11/H1002/10. To protect participants’ identities, identifiers are used below, with N referring to a nurse and D to a dietitian.

## Results

The 18 staff members interviewed were all women who came to the trial with extensive diabetes clinical experience, having specialised in the field of diabetes care for between six and 30 years (mean: 19 years). All staff described undertaking work on the trial on a part-time basis alongside their routine clinical hospital-based duties, with trial funding having been used to buy out their time. In keeping with the broader trials literature (see above), staff highlighted a plethora of logistical and practical issues which had made recruitment challenging and other aspects of the trial hard to deliver. However, alongside these practical issues, staff often talked spontaneously and at length about how their involvement in the trial had affected them emotionally. Indeed, in many cases, as we will highlight below, emotional challenges arose directly from the practical demands of undertaking work on the trial. As well as having to manage their emotions, many staff described how they had had to undertake emotion work on their colleagues and also on patients. As our interviews also made apparent, the emotional challenges arising from trial work could be heightened by virtue of staff having to maintain ongoing clinical relationships with patients after the trial. Below, we consider these findings in more depth before going on to describe how staff felt they could be better supported in the future to deliver and address the emotional elements of trial work.

### Practical aspects of trial work

In line with the trials literature described above, all the staff described a number of practical issues and considerations involved in delivering the REPOSE trial. This included a lot of seemingly mundane non-clinical tasks which, as they pointed out, nevertheless took up a lot of their time, such as phoning patients, making and changing appointments, chasing non-attenders and completing ‘the endless forms, there did just seem to be so many bits of paper’ (N8). However, where the practical aspects and demands of trial delivery work came particularly to the fore was when staff shared their experiences of recruitment. While some centres had found recruitment easier than others, virtually all staff described trial recruitment as having been harder, and as having required more time and effort, than originally anticipated. This was due in part to the complex nature of the randomisation process and the use of a group-based intervention which, as N2 described, had meant that:

‘As soon as we’ve got courses one and two filled, you’re straight onto recruiting for courses three and four … we didn’t really think about how intensive the recruitment period would be.’ (N2)

Recruitment presented particular challenges for staff working in established DAFNE centres as these did not tend to have large numbers of patients on course waiting lists whom staff could invite to participate in the trial. Hence, in these centres, staff reported having had to develop a diversity of recruitment strategies from the outset, including: recruitment evenings, mailshots, liaising with GPs in order to receive referrals, searching electronic databases to identify potential recruits and advertising in local media. In addition, when these initial recruitment strategies did not yield as many trial recruits as staff had hoped for, they described how they had had to invest in even more labour intensive strategies; in D1’s case by phoning patients individually after they had not responded to written invitations:

‘… it was quite hard, especially when you sent out all those mailshots and you weren’t getting anybody, any response as well, you were having to phone people and not getting anybody so that was probably quite stressful, dealing with all those people.’ (D1)

As staff further observed, such recruitment strategies had also required them to work beyond their usual hours in order to try and catch people at home, thereby blurring the usual public/private boundaries of hospital-based work:

‘I couldn’t sort of set aside specific days to do it, it would end up sort of eating into the evenings and/or coming in very early.’ (N2)

### Emotional aspects of trial work

#### Recruitment: pressures to meet trial targets

‘We were worried we wouldn’t meet the numbers.’ (D3)

‘There was a panic coming towards the deadline, have you got enough numbers?’ (N5)

The practical aspects of trial work were not just experienced as physically demanding and time consuming, as the above quotations suggest, staff also described their recruitment experiences as having been very emotionally demanding or, as D6 put it, ‘an absolute nightmare’. A key reason for this, as some staff pointed out, was that while their centres had had trial-specific time funded (for example, to buy them and their colleagues out from some of their routine clinical work), recruitment had been much more time-consuming than originally anticipated. As a consequence, staff described how they had had to juggle trial recruitment work alongside their routine clinical work in ways which they had found stressful and emotionally taxing:

‘I am still a clinical nurse and although I’ve been given two days for doing the trial - I’ve got to say that’s been challenging even fitting those two days in because the work involved is much more than two days’ worth of work. And we’re at a time where we’ve got staffing pressures and to be honest, fitting everything in with the staffing pressures has been really challenging and really quite stressful at times.’ (N1)

In addition, even when staff were not directly involved in recruitment, they often reported having found it distressing seeing their colleagues struggling with, and worrying about, recruitment. As a consequence, staff described how they had taken time out of their clinical roles to offer encouragement and emotional support:

‘… [she]’s just found it really, really hard, and it’s been awful watching her struggle with recruitment, thinking, ‘oh poor you’ has just been really hard.’ (N6)

‘… you know, I’ve seen [name of centre principal investigator] pulling his hair out on a regular daily basis… there was a lot of stress around about that time, so I tried to be quite laid back throughout, you know, reassuring them that “we will get the numbers”, I tried to be confident we would get there.’ (N3)

All staff also reflected on their experiences of receiving regular updates from the CTU via emails and mailshots on how the different centres were progressing with recruitment and reaching their targets. In general, staff described this input as motivational and as having helped to keep them on track: ‘it definitely filtered down to us, we had constant reminders from central office, so that’s been really good.’ (N2). However, some staff also described how it had made them feel ‘a little bombarded and badgered’ (N12), especially those, such as N6, who belonged to centres which, by virtue of comparisons drawn with others, appeared to be underperforming:

‘… we did get the newsletters which showed in graph form who was getting their targets and who wasn’t, so we knew that we were under pressure! And I knew that, well we knew that anyway because we were struggling to get recruits […] And so, yeah we knew that we were, you know, bottom of the table as far as recruitment goes.’ (N6)

Alongside the emotional stresses of recruiting into the trial and reaching trial targets, some staff also highlighted the emotional challenges which could arise from what they perceived as the conflicting priorities of their research roles and clinical responsibilities. Staff, for instance, described how they had worried about placing undue pressure on patients to participate in the trial, due to the pressure they themselves were under to reach their targets, and the potential impacts that this might have had on their clinical relationships and on themselves:

‘And you don’t want to hound people. I felt with this research, they’re volunteers, we shouldn’t be hounding them onto this course. And that was my concern… I wanted to still be seen as a nurse. Because I’ve got to go back and have relationships with these people later on, so I want to be seen as a nurse and not as the research nurse.’ (N1)

As a result, staff, such as D2, described having had to use their own intuition and judgment to decide when to stop and leave a patient alone:

‘… we will try to chase everybody up for the, you know, as much as we can, but, there’s certain people after you’ve contacted them so many times obviously you get the message they don’t want to be involved.’ (D2)

#### Dealing with patients’ emotions at randomisation

‘Some people did have quite strong opinions, but you tended not to find out about their opinions until they’d been randomised’ (N6)

‘There’s sort of, sometimes an intake of breath, and you say, you know “I hope you are not disappointed if it’s not the course you want.”’ (D4)

As the above quotations suggest, where the emotional aspects of trial work particularly came to the fore was when staff shared their experiences of contacting patients to notify them about their randomisation outcome. This task was normally done on the phone, and most patients were described as having been positive or philosophical about their treatment allocation. However, staff in virtually all centres recounted instances when they had had to emotionally manage patients who, despite claiming to have had no treatment preference when they gave informed consent (this having been a requirement to take part in the trial), could be ‘very disappointed and angry’ (D2), ‘gutted’ (N1) and ‘really mad’ (N6) when they learned of the outcome. Not only did some patients question and challenge staff about the outcome of randomisation: ‘occasionally, they’ll say to us, “oh do you randomise… and they wouldn’t have been happy if it had been us, so we did have to explain that it is completely, you know, out of our control’ (D4), such patients were also described as having needed a lot of comfort and reassurance in order to come to terms with news of their treatment allocation:

‘… she was upset that she got the pump and she didn’t think she would be able to handle it. So it meant the other educator and I just spent some time just talking with her and saying, “We think you’re going to be alright and we’re going to help you through it”, and so it just took a bit, quite a lot more time to calm her, I think, and reassure her and just show her.’ (N6)

While, in the above example, emotion management had been required after the female patient discovered that she had been randomised to the trial’s pump arm, most staff described realising, early into the trial, that negative reactions were most likely to occur when patients discovered they were not going to get a pump. As staff described, it was not until this point in the trial that some patients confessed to them that, despite having stated they had no treatment preference when they were recruited, they had actually agreed to participate in the hope of getting a pump. This realisation, as staff went on to suggest, had enabled them to develop strategies to pre-empt or manage potential disappointment and anger, wherein: ‘we tried to make it [randomisation to MDI] very positive’ (N2). This included N9 who talked about how she had encouraged patients to share their emotions and talk freely before going on to present their randomisation to the MDI arm in a more positive light in order to foster their motivation and commitment to remain in the trial:

‘I acknowledged it - or I allowed them to acknowledge that they were disappointed, you know, I said “how do you feel about that?” and they said “oh well, I was hoping to get a pump.” And then I would be positive, I said “well, you know, you’re going to get structured education, you’ll benefit from this, you know, you’re going to be in a group, there’s so many positive things about it, blah-blah-blah.”’ (N9)

Staff, such as N12, also described how they had ‘talked up’ the DAFNE MDI course to ease patient disappointment and anger by pointing out that, in routine clinical situations, people had to attend this type of course before (at a later stage) becoming eligible for a pump:

‘I told them that the way that the guidelines are at the moment anyway for being put on a pump, they would have to do some kind of structured education, so if in the future it’s, they, they fit into that criteria and they’ve already done the DAFNE then that’s only of benefit to them.’ (N12)

#### Managing one’s own emotions

As well as undertaking emotion work to address or pre-empt patients’ upset and disappointment, staff also described how they had had to manage their own emotions at various stages during the trial, a critical point being when they had notified patients of the outcome of randomisation. At this stage, some patients decided to withdraw and, given the time and effort they had already invested in them and in reaching recruitment targets, staff described how they had experienced these withdrawals as ‘disheartening’ (D1) and as a source of frustration:

‘And I think that’s the frustration, is you can get them to sign the consent form, you can get them recruited, you can phone them the week before and say, “Are you coming?” you can see them at the pre-assessment, but they still don’t turn up. And there’s absolutely nothing that you can do about that.’ (D6)

Indeed, staff were acutely aware of their ongoing clinical relationship with some patients who withdrew or dropped out of the trial and that they could not convey their disappointment and anger to such patients. Hence, in these kinds of situations, staff described having had to make effort to manage or mask their own emotions:

‘I phoned one guy and as I was talking to him, telling him about the course, he said “I will probably not be able to do that course”, and in my head I was thinking “right is that really true or you just don’t want the pump or whatever?”’ (D4)

In addition to having to deal with the frustration of patients withdrawing from the trial, some staff also highlighted instances when they themselves had felt disappointed when particular patients had not been randomised to the treatment arm which, in their clinical judgment, they had felt would have been in their best interests (typically a pump). This included N1, a self-proclaimed ‘pump enthusiast’, who described this aspect of her trial work as having been emotionally taxing, as well as having required her to undertake impression management [42] with patients in order to mask her own ambivalence:

‘There was definitely disappointment. One of the girls was crying, she was in tears and that was upsetting for me. Because I, to be honest, I really wanted to give her that pump. I knew that some of them really did want pumps…. It, it was, it was quite stressful because, as I say, it, for me it was about presenting a balanced view to somebody I felt would have done better on a pump.’ (N1)

#### Staffs’ need for support

Some staff described how they had had reservations about participating in the trial due to their carrying heavy workloads and/or their concerns about their centre not having a long patient waiting list which could be drawn upon for recruitment purposes. These staff, including N11 below, described how they would have liked and benefitted from being more involved in discussions and from being given more information and reassurance before the final decision to take part in the trial had been made:

‘Our consultant had agreed without talking to us […] that caused us massive anxieties, there was lots of meetings with our, our line manager and our consultant and … us saying “No we’re not doing it” and them saying “Yes you are”. Lots of … lots of meetings before we kind of finally felt happy to go ahead.’ (N11)

In addition, many staff described how, with hindsight, they would have benefitted from more resources to undertake recruitment; specifically, more administrative input to ‘help chase people up and stay on top of the paperwork’ (D2). As such staff further speculated, having this type of input might have also helped to ease the emotional pressure and worry they had experienced whilst attempting to meet trial targets. Some staff also described having benefitted, or how they would have benefitted, from using some of their trial income to employ research nurses. This was not only to reduce pressures on their own time, but also because these professionals were perceived as being less likely to experience role conflicts than themselves:

‘they are used to the whole idea of talking people through patient information leaflets and explaining the pros and cons … and they don’t have to care for them [patients] after the trial is over.’ (N3)

As well as needing more practical input, staff also highlighted a need for more emotional support. While some staff did describe benefitting from receiving this kind of support from colleagues, they also pointed out that emotional support had mostly been informal and, hence, delivered and received on an *ad hoc* basis. For this reason, staff suggested that opportunities to receive peer and/or team emotional support could be formalised in future trials; for example, through ‘proper debriefing sessions’ (D4) and pre-scheduled meetings.

In addition, staff described a need for support ‘dealing with patients that were disappointed and upset about not getting the treatment arm they wanted’ (N6). This was because they had often found themselves having to adopt psychological and emotional management roles for which they had felt unprepared: ‘I mean you were counselling them on the phone, you were definitely counselling them and we had to learn to do that on the hoof’ (N1). Hence, some staff suggested that they would have benefitted from input, training and support from experienced psychologists and counsellors in order to learn how to undertake effective emotional management of patients who could be confrontational, angry and/or upset:

‘I think that we, as educators meeting that disappointment, we could have possibly been supported a bit more… But as I say, you’ve got one person being really angry, one person, one girl crying her eyes out and, I mean, you know, she, she… as I say, I just found that really upsetting and, you know, what can I say to her to make it better, she just so wants that treatment? And, I do think we could have done to have discussed that more, the psychology, there was, there wasn’t enough… there was a lot of clinical stuff in doing the trial but the psychology to me that’s underpinned a lot of what we’ve done, we could have done a bit more on that.’ (N1)

## Discussion

This study explored the experiences and views of staff involved in a clinical trial investigating two methods of insulin treatment delivery. What staff’s accounts have served to highlight is that, alongside the physical and logistical challenges involved in trial delivery [14–16], and the ethical challenges which can arise from staff having to balance research roles with clinical responsibilities [23–25], trial work can also involve attendant emotional challenges [30, 31] and require staff to undertake emotion work. Such work may not only require staff to pre-empt and manage patients’ emotions, such as their potential anger or disappointment on hearing the outcome of randomisation, it may also entail staff managing their own emotions and those of colleagues. Like the emotional labour uncovered by James in her study of hospice nurses [34] and first reported by Hochschild in a study of flight attendants [33], this emotion work is arguably an invisible and hitherto underreported aspect of trial work. It is, however, a kind of work which deserves more consideration and attention to help ensure staff are appropriately supported to undertake trial work in the future.

Since the early work undertaken by James [34] and others [35, 36], there has been a growing recognition of the importance, yet undervalued nature, of emotional labour in nursing and midwifery work. Indeed, a now extensive and diverse range of studies have shown this kind of labour to be an integral aspect of nursing and midwifery work, spanning a wide range of disciples and sub-specialties, including: mental health nursing [43], end of life nursing [34, 44], dementia care [45] and gynaecology [46, 47]. As this body of work, when brought together, has also served to highlight, and in keeping with the findings reported in this paper, emotional labour can be a complex and multi-faceted task. Specifically, it can entail staff resourcefully employing empathy and ‘emotional intelligence’ [48] to understand patients’ perspectives and provide them with emotional support [46–48], as well as offering similar support to colleagues [49]. It can also require staff to emotionally manage themselves in order to present an appropriate emotional response and persona to others [46, 50], and it can extend from public and workplace settings into domestic and private spheres [45]. As some studies have further served to highlight, the emotional labour undertaken by staff can also be affected by organisational features and constraints, such as ward routines and requirements to follow strict protocols [34, 51]. The impact of organisational features was also apparent in our study in which we found that staff members’ experiences of stress and the attendant emotional work undertaken could be affected by whether their centre was a new or established DAFNE centre with a long or short patient waiting list.

As indicated earlier in this paper, a common agenda of the nursing and midwifery research has been to raise awareness of the importance and highly skilled nature of the emotion work undertaken by staff. It has also been to highlight the inadequacy of current staff preparation and training [47, 48], which has mostly been informal and learnt on the job [34, 47, 52]. Hence, these studies have called for better and more formalised training and support to be offered to staff to undertake effective emotion work [34, 43, 44, 48]. Such a call, it could be argued, should be extended to staff working on clinical trials [30, 31]. However, it should also be kept in mind that, despite the rapid growth of scholarly activity within the field of emotions, it has had disappointingly little impact on everyday nursing and midwifery practice [49, 53]. This situation has been attributed to emotional work being marginalised by the dominant emphasis on clinical outcomes, cost containment and evidenced-based practices [53, 54], and has led to calls for pragmatic and realistic approaches to be developed [54].

In developing pragmatic and realistic approaches which could be used in trial work, a number of potential avenues could be considered. One would be to check that sites interested in participating in a clinical trial have the capacity to deliver the requirements of the study and have in place an infrastructure to support implementation. Some of the staff we interviewed indicated that they had felt ill-prepared for the work involved in undertaking recruitment and would have liked to have been more involved in decisions about taking part in the trial. Hence, we would recommend that site checks should entail discussion with all front-line staff who would be involved in trial recruitment and delivery, as well as with principal investigators.

Another potential avenue to consider would be to develop and use effective approaches for pre-empting and preventing potential disappointment about treatment allocation (and, hence, the need for staff to undertake emotion work on patients and themselves). This could potentially be achieved by improving patient and staff understandings of trial designs, in line with others’ recommendations [8, 20]. However, as qualitative research undertaken with patients and/or their proxies (for example, the parents of newborn infants) has served to highlight, this may not necessary be an easy task [7–9]. This is because, even when people are given clear and accessible information (as happened in the REPOSE trial) and are able to accurately recall it, they can still find it very difficult to grasp and understand concepts such as equipoise and randomisation. Hence, provision of information to patients in isolation is unlikely to be sufficient [8]. To address this problem, promising work has been undertaken by Donovan *et al*. [55] who used a complex intervention, informed by ongoing qualitative findings, to improve training given to recruiting staff (for example, through individualised feedback following review of audio-recorded recruitment appointments and through updating of training materials throughout the trial). This intervention was shown to have a positive impact on patients’ understandings of the trial, informed consent and, hence, acceptance of treatment allocation [55]. The benefits of staff training have also been highlighted in another study [12]. In this, staff described how they had been able to undertake trial work in an unbiased way after receiving training to raise their awareness of their own treatment preferences and how these might impact on trial delivery practices. Hence, this has led to a recommendation that similar, proactive training be incorporated into future trials [12].

In addition, we would recommend, like others, that trial staff would benefit from increased resourcing and administrative support [15]. Staff might also benefit from greater provisioning of experienced research nurses to undertake recruitment. Indeed, use of such staff might not only lessen trial staff’s workloads, but also the ethical issues and attendant emotional challenges which can arise when conflicts occur between clinical and research roles [25]. However, given these kinds of interventions can be expensive, other potentially more cost-effective and easy to implement alternatives may need to be considered. To this end, and drawing upon the ideas, suggestions and positive experiences reported by staff who took part in the REPOSE trial, future trials could include regular sessions for frontline staff, perhaps led by the principal investigator or other senior team member, with local psychological support. These could be used to enable and encourage staff to discuss emotionally challenging issues, seek input and emotional support from colleagues and to critically reflect on experiences [47]. Depending on the size of the trial team within particular centres, these sessions could be centre specific or held between sites. In addition, staff could benefit from being able to access a psychologist or counsellor on a one-to-one basis, when required, to discuss complex emotional and ethical issues, seek advice on how these could be addressed and receive targeted support for themselves. We would also recommend that staff are provided with training to help them to develop and use effective strategies for dealing with potential patient disappointment or anger following notification of treatment allocation. This could comprise role-play of hypothetical scenarios before the trial begins and in anticipation of problems and challenges arising, and use of actual cases occurring during the trial to learn from ongoing experiences and share good practice (see also Donovan *et al*. [55]). To deliver these kinds of interventions, we would also recommend that appropriate funding is included in future grant applications. In addition, we would recommend that such interventions should be evaluated for effectiveness and cost-effectiveness.

In making these recommendations, it is important to consider that they are informed by findings from an exploratory study of a relatively small number of staff working on one particular trial, all of whom were women and all of whom were either nurses or dietitians. This may limit the generalisability of the findings, not least given Donovan *et al*.’s observation that different ethical issues and attendant emotional challenges may arise for different categories of trial staff; in their case, doctors versus nurses [30]. It should also be taken into account that emotion work is stereotypically considered women’s work [34] and, hence, one which female staff members are thought to be particularly well placed to undertake. Therefore further research involving different categories of staff working on different kinds of trials would be beneficial; this could also include observational as well as interview methodologies. These reflections notwithstanding, we would suggest that our findings and recommendations do have a potential resonance and relevance for staff working on other types of clinical trials, especially those which, like REPOSE, are non-blinded and, hence, where there is potential for patient and staff disappointment about randomisation outcomes. It is also pertinent to consider that REPOSE is a relatively uncontroversial trial in so far as, while it deals with a chronic health condition, it does not involve patients at the end of their lives or those who might be motivated to use a trial to access potentially lifesaving treatments. Hence, it is possible that the emotional issues highlighted in this paper could be even greater in trials involving more controversial treatments or with greater morbidity and/or mortality implications.

## Conclusions

To improve the experiences of staff working on clinical trials and help promote a successful trial, this paper calls for more attention to be paid to the emotional aspects of trial delivery. Indeed, the complexity of emotional experiences and support needs described by staff working on this relatively uncontroversial trial suggests that emotional labour is an important issue for trial managers, research teams, funding bodies and others to consider when developing, funding and supporting future trials.

## Abbreviations

CTU: Clinical Trials Unit
D: Dietitian
DAFNE: Dose Adjustment for Normal Eating
GP: General Practitioner
MDI: Multiple Daily Injections
N: Nurse
NHS: National Health Service
REPOSE: Relative Effectiveness of Pumps Over MDI and Structured Education
RCT: Randomised Controlled Trial.
